# Computational Approaches for the Discovery and Development of Pharmacologically Active Natural Products

**DOI:** 10.3390/biom11050630

**Published:** 2021-04-23

**Authors:** José L. Medina-Franco

**Affiliations:** DIFACQUIM Research Group, Department of Pharmacy, National Autonomous University of Mexico, Mexico City 04510, Mexico; medinajl@unam.mx; Tel.: +52-55-5622-3899

Natural products continue to be a significant source of active compounds. Natural products from different sources have provided many molecules approved for clinical use or used as starting points for hit-to-lead optimization programs [1]. Similarly, natural products have inspired the synthesis and development of biologically active molecules. Indeed, it is also well known that nature has selected and optimized chemical structures to produce chemical scaffolds and compounds enriched with biological function over millions of years. However, identifying and developing pharmacologically active natural products efficiently and systematically is challenging. Hurdles in natural products research include challenges in isolation and purification procedures, minimal available amounts of lead compounds, difficulty in synthesizing natural products with high structural complexity, and associated synthesis scale-up issues. Moreover, for drug discovery applications, caution should be taken with compounds that have been designed by nature for defense and are toxic. As such, one can expect that not all natural products have a beneficial effect on health. However, the considerable success of using natural products to produce bioactive compounds or bioactive mixtures has inspired the preparation of synthetic molecules that have become drugs approved for clinical use. To help overcome these challenges, a broad range of computational approaches have evolved in recent years, and the number of applications of computational approaches to improve and accelerate natural product-based drug discovery is increasing [2]. 

Computational methods include molecular modeling, chemoinformatics, bioinformatics, artificial intelligence, machine learning, and other approaches that involve computer-aided drug design [3]. One of the key contributions of chemoinformatics and computational approaches, in general, has been provided by the increasing availability and development of compound databases in the public domain, including natural product databases [4]. The latter has boosted applications of computational methods to natural product-based drug discovery. 

This Special Issue aims to discuss advances in computationally driven advances and applications of different methods to identify and develop pharmacologically active natural products. This Special Issue includes research papers and a review article from more than 85 scientists from more than 10 countries worldwide. The contributions show the application of a wide range of in silico methods applied to natural product research. Computational methods used in different contributions involved a broad range of disciplines, including chemoinformatics, bioinformatics, molecular modeling, data mining, and data visualization. The research papers showed applications in several important areas, such as identifying candidate compounds for treating diverse diseases, including neglected tropical diseases and COVID-19, which, in 2020, marked a hallmark in health, everyday life, and research. Contributions also included the development and computational analysis of natural product databases. The review paper published in this Special Issue focused on the applications of chemoinformatics to characterize pharmacologically active natural products, leading to the proposal of a sub-discipline of so-called “natural products informatics.”

Considering the current era of artificial intelligence and the overall increased application of computer-aided drug design methods to produce natural products and food chemicals, we emphasize the need to use these approaches rationally. We encourage students and newcomers to the computational field to not go along with fashion, but to use any (computational and experimental) approaches for the right reasons, and not just because they are easy to use and many are easily accessible. Thus, we encourage the community to learn computational techniques as thoroughly as possible and to distinguish artificial intelligence from common sense.

Overall, the manuscript in this Special Issue demonstrates examples of the advances in computational approaches for the study, discovery, and development of pharmacologically active natural products. It is expected that the contributions by all the authors that kindly contributed to this issue contribute to advancing the field of natural product informatics [5]. Finally, on behalf of all the authors that contribute to the issue, we expect that it serves as a source of valuable information and inspiration to newcomers to the field and students to continue advancing the discovery of pharmacologically active compounds from natural sources.

## References

[1] Atanasov A.G., Zotchev S.B., Dirsch V.M., Orhan I.E., Banach M., Rollinger J.M., Barreca D., Weckwerth W., Bauer R., Bayer E.A. (2021). Natural products in drug discovery: Advances and opportunities. Nat. Rev. Drug Discov..

[2] Chen Y., Kirchmair J. (2020). Cheminformatics in Natural Product-based Drug Discovery. Mol. Inf..

[3] Gasteiger J. (2020). Chemistry in Times of Artificial Intelligence. ChemPhysChem.

[4] Madariaga-Mazón A., Naveja J.J., Medina-Franco J.L., Noriega-Colima K.O., Martinez-Mayorga K. (2021). DiaNat-DB: A molecular database of antidiabetic compounds from medicinal plants. RSC Adv..

[5] López-López E., Bajorath J., Medina-Franco J.L. (2021). Informatics for Chemistry, Biology, and Biomedical Sciences. J. Chem. Inf. Model..

